# FKBPL and SIRT-1 Are Downregulated by Diabetes in Pregnancy Impacting on Angiogenesis and Endothelial Function

**DOI:** 10.3389/fendo.2021.650328

**Published:** 2021-06-02

**Authors:** Abdelrahim Alqudah, Kelly-Ann Eastwood, Djurdja Jerotic, Naomi Todd, Denise Hoch, Ross McNally, Danilo Obradovic, Stefan Dugalic, Alyson J. Hunter, Valerie A. Holmes, David R. McCance, Ian S. Young, Chris J. Watson, Tracy Robson, Gernot Desoye, David J. Grieve, Lana McClements

**Affiliations:** ^1^ The Wellcome-Wolfson Institute for Experimental Medicine, School of Medicine, Dentistry and Biomedical Sciences, Queen’s University Belfast, Northern Ireland, United Kingdom; ^2^ Department of Clinical Pharmacy and Pharmacy Practice, Faculty of Pharmaceutical Sciences, The Hashemite University, Zarqa, Jordan; ^3^ Centre for Public Health, School of Medicine, Dentistry and Biomedical Sciences, Queen’s University Belfast, Northern Ireland, United Kingdom; ^4^ Royal Jubilee Maternity Hospital, Belfast Health and Social Care Trust, Northern Ireland, United Kingdom; ^5^ Medical Faculty, University of Belgrade, Belgrade, Serbia; ^6^ Department of Gynaecology and Obstetrics, Medical University of Graz, Graz, Austria; ^7^ Clinic of Obstetrics and Gynecology, Clinical Centre of Serbia, Belgrade, Serbia; ^8^ Royal Victoria Hospital, Belfast Health and Social Care Trust, Northern Ireland, United Kingdom; ^9^ School of Pharmacy and Biomolecular Sciences, Irish Centre for Vascular Biology, RCSI University of Medicine and Health Sciences, Dublin, Ireland; ^10^ School of Life Sciences, Faculty of Science, University of Technology Sydney, Sydney, NSW, Australia

**Keywords:** FKBPL, SIRT-1, GDM, pregnancy, angiogenesis, Diabetes, Trophoblasts, endothelial cells

## Abstract

Diabetes in pregnancy is associated with adverse pregnancy outcomes including preterm birth. Although the mechanisms leading to these pregnancy complications are still poorly understood, aberrant angiogenesis and endothelial dysfunction play a key role. FKBPL and SIRT-1 are critical regulators of angiogenesis, however, their roles in pregnancies affected by diabetes have not been examined before in detail. Hence, this study aimed to investigate the role of FKBPL and SIRT-1 in pre-gestational (type 1 diabetes mellitus, T1D) and gestational diabetes mellitus (GDM). Placental protein expression of important angiogenesis proteins, FKBPL, SIRT-1, PlGF and VEGF-R1, was determined from pregnant women with GDM or T1D, and in the first trimester trophoblast cells exposed to high glucose (25 mM) and varying oxygen concentrations [21%, 6.5%, 2.5% (ACH-3Ps)]. Endothelial cell function was assessed in high glucose conditions (30 mM) and following FKBPL overexpression. Placental FKBPL protein expression was downregulated in T1D (FKBPL; p<0.05) whereas PlGF/VEGF-R1 were upregulated (p<0.05); correlations adjusted for gestational age were also significant. In the presence of GDM, only SIRT-1 was significantly downregulated (p<0.05) even when adjusted for gestational age (r=-0.92, p=0.001). Both FKBPL and SIRT-1 protein expression was reduced in ACH-3P cells in high glucose conditions associated with 6.5%/2.5% oxygen concentrations compared to experimental normoxia (21%; p<0.05). FKBPL overexpression in endothelial cells (HUVECs) exacerbated reduction in tubule formation compared to empty vector control, in high glucose conditions (junctions; p<0.01, branches; p<0.05). In conclusion, FKBPL and/or SIRT-1 downregulation in response to diabetic pregnancies may have a key role in the development of vascular dysfunction and associated complications affected by impaired placental angiogenesis.

## Introduction

Hyperglycaemia is one of the most common pregnancy complications, affecting one in six pregnancies (1). Hyperglycaemia in pregnancy could be due to: i) type 2 diabetes mellitus (T2D) characterised by hyperglycaemia, insulin resistance and some insulin deficiency (2), ii) type 1 diabetes mellitus (T1D) - an autoimmune disease where the body’s immune system attacks insulin-secreting pancreatic β islet cells leading to insulin deficiency (3), and iii) gestational diabetes mellitus (GDM) that occurs only in pregnancy characterised by impaired insulin function due to the production of hormones by the placenta leading to insulin resistance. GDM is usually a transient condition during pregnancy, which resolves immediately after delivery of the baby, although in some women GDM continues as T2D beyond pregnancy (4, 5). The vast majority (~86%) of hyperglycaemia cases in pregnancy are attributed to GDM (6).

Gestational hypertension and preeclampsia are the leading causes of morbidity and mortality in pregnancy (7), and in the presence of hyperglycaemia, the risk of these conditions is increased up to 4-fold (8). Indeed, elevated fasting blood glucose and HbA1c have been associated with increased risk of preeclampsia in pregnant women with GDM, T1D or T2D (9–11). In addition, hyperglycaemia during pregnancy can lead to several other adverse neonatal complications, including the large for gestational age (LGA) foetus, shoulder dystocia, respiratory distress syndrome and neonatal hypoglycaemia (12). Hyperglycaemia in pregnancy also increases the risk of perinatal death and stillbirth by at least two- to three-fold, particularly in the presence of T1D and T2D compared to healthy pregnant controls (13, 14). Women who develop GDM in pregnancy have a 50% increased risk of developing GDM in a subsequent pregnancy, and a 10-fold increased risk of developing T2D within 10 years following pregnancy (15). In addition, children born to mothers with GDM or pre-existing diabetes are at higher risk of developing obesity, T2D and cardiovascular disease during their life-time (16–18). Despite clear epidemiological link between diabetes in pregnancy and both short- and long-term pregnancy complications, the mechanisms of these associations are still poorly understood.

The placenta is a highly vascularised organ, which facilitates adequate oxygen and nutrient transfer from the mother to foetus as well as removal of foetal waste products through maternal circulation (19). Placental development and growth are tightly regulated by angiogenic factors, such as vascular endothelial growth factor (VEGF), placental growth factor (PlGF), and their receptors, which stimulate angiogenesis throughout pregnancy in a controlled manner (20). However, in pregnancies complicated by diabetes, pre-existing endothelial dysfunction and aberrant angiogenesis can lead to hyper-vascularisation of the placenta (21). This process of overstimulated placental angiogenesis can lead to impairment in the integrity of both the maternal and foetal vascular system as well as increased peripheral vascular resistance and maternal hypertension (22).

FK506-binding protein like (FKBPL), a divergent member of the immunophilin protein family, is a key anti-angiogenic protein that also regulates glucocorticoid receptor signalling; the mechanisms involve HSP90, CD44 and Notch (23–26). Both of these key functions regulate metabolism and vascular health (27, 28), therefore suggesting a potentially important role for FKBPL in diabetes and the associated vascular dysfunction. Sirtuin-1 (SIRT-1), an NAD+ dependant deacetylase, also has a critical role in endothelial cell metabolism and function. In diabetes, SIRT-1 is downregulated, which is often associated with endothelial dysfunction (29, 30). Previous studies have also demonstrated that FKBPL knockout mice were embryonically lethal, highlighting its vital role in embryonic development. Whilst heterozygous FKBPL knockdown mice developed normally, the vasculature appeared leaky with compromised integrity, implicating FKBPL with a key function in endothelial function (31). FKBPL has also been shown to inhibit tumour angiogenesis; this has led to the development of therapeutic peptides for the treatment of solid tumours (32, 33). In preeclampsia, FKBPL and its target protein, CD44, have demonstrated their predictive and diagnostic biomarker potential, likely reflective of the pathogenesis of preeclampsia and placental hypoxia (34, 35). However, FKBPL’s role in the context of diabetes in pregnancy has not yet been investigated. Therefore, in this study, placental expression of FKBPL in conjunction with SIRT-1, PlGF and VEGF-R1 was investigated in human samples from pregnant women with pre-existing diabetes (T1D), and GDM. FKBPL and SIRT-1 expression was also assessed in ACH-3P first trimester trophoblasts in response to high glucose and varying oxygen concentrations relevant to placental development (35). The importance of FKBPL's role in endothelial cell angiogenesis in both low and high glucose environments was demonstrated.

## Methods

### Placental Samples

Pregnant women with GDM and T1D as well as healthy pregnant controls, provided written informed consent as part of their recruitment to the PREDICT study at the Royal Jubilee Maternity Hospital (36). Ethical approval for this project was obtained from the NHS Health Research Authority (ORECNI, 14/NW/1222) and the School of Medicine, Dentistry and Biomedical Sciences (Queen’s University Belfast). Clinical characteristics were recorded [age, BMI, gestational age (GA), mode of delivery (MOD), parity and foetal sex], systolic blood pressure (sBP) and diastolic blood pressure (dBP) were measured according to the National Heart Foundation of Australia protocol using automated devices as previously described (37) in both the left and right arm of each patient on two occasions during the first trimester. Women with T1D were on insulin whereas we did not obtain information on medication from women with GDM as they were recruited before any treatment was initiated. Healthy controls did not take any medication.

Placental sections were collected from women with T1D, GDM or healthy pregnancies after the baby was delivered. All slides were stained by standard haematoxylin and eosin (H&E) procedure. Following staining, placental morphology and pathophysiology was assessed by a pathologist, DO. The parameters analysed included placental maturity, vascularisation and branching of chorionic villi by characterising the structure of chorionic villi, area of terminal villi covered by blood vessels, presence or absence of calcifications and syncytial knots. The distribution of syncytial knots was quantified by counting the number of syncytial knots per 100 terminal villi.

### Immunofluorescence Staining

Placental section slides were prepared by the Northern Ireland Biobank (38) using fresh tissue before being subjected to immunofluorescence staining for FKBPL (Cat. no.: 10060-1-AP; Proteintech, UK), SIRT-1 (Cat. no.: ab110304; Abcam, UK), PlGF (Cat. no.: ab180734, Abcam, UK) and VEGF-R1 (Cat. no.: AF321, R&D systems, USA). Tissue slides were imaged using a Leica DMi8 fluorescence inverted microscope using the same magnification (20x) and exposure. Analysis was performed using Image J software (NIH, US) by selecting six random fields of view per section and measuring the intensity, with the assessor blind to patient group. Protein expression was quantified as previously described (39).

### Cell Culture

ACH-3P cells were kindly provided by Professor Gernot Desoye (Medical University of Graz, Austria). Briefly, immortalised choriocarcinoma cells, AC1-1 cells, and primary trophoblast cells isolated from the first trimester placenta were fused (40), to form a unique ACH-3P cell line as previously described (41). ACH-3P cells were maintained in Hams F-12 medium supplemented with 10% FBS and all experiments were carried out at Medical University of Graz. Short Tandem Repeat (STR) DNA profiling analysis was performed using PowerPlex 16 HS System (Promega, UK) for cell authentication. ACH-3P cells were exposed to high glucose (25 mM) or mannitol (20 mM + 5 mM D-glucose) as an osmolality control under different oxygen concentrations (21%, 6.5%, and 2.5%) for 24 h. This was based on previous experimental design where the least cell death was observed (35). Protein was then extracted for downstream protein expression analysis by Western blotting.

Human Umbilical Vein Endothelial Cells (HUVECs) (ATCC, USA) kindly donated by Dr Andriana Margariti (Queen’s University Belfast) were maintained in MV2 endothelial cell growth media (PromoCell, Germany) supplemented with low serum growth supplement containing the following: 5% foetal calf serum (FCS), epidermal growth factor 5 ng/ml, basic fibroblast growth factor (FGF) 10 ng/ml, insulin-like growth factor 20 ng/ml, VEGF 0.5 ng/ml, ascorbic acid 1 μg/ml and hydrocortisone 0.2 μg/ml.

All cells were maintained at 37°C in a humidified atmosphere with 5% CO_2_.

### Tubule Formation Assay

HUVECs were transfected with a FKBPL overexpressing plasmid (pFKBPL; Sino Biological, USA) or empty vector plasmid (pCMV3; Sino Biological, USA) for 24 h before being plated in Matrigel (Corning, UK). HUVEC (7x10^5^) were stained with calcein (2µg/ml; Thermo Fisher Scientific, UK) prior to being seeded on phenol red free conditions reduced-growth factor Matrigel under high glucose conditions (30mM D-glucose) or mannitol (25 mM + 5 mM D-glucose) for 24 h. Tubule formation was imaged by randomly capturing six images per well using a DMi8 inverted florescence microscope. The number of branches and junctions were quantified using Image J software (NIH, USA).

### Western Blotting

HUVEC and ACH-3P protein lysates were harvested using RIPA buffer (SantaCruz Biotechnology, USA) supplemented with protease and phosphatase inhibitor cocktails (Roche, UK) and subjected to Western Blotting. FKBPL (Proteintech, UK, cat# 10060-1-AP) and SIRT-1 (Abcam, UK, cat# Ab110304) antibodies were used at 1:1000 dilution. Appropriate HRP-linked secondary antibodies were used at 1:10 000 before chemiluminescent was applied and blots imaged as previously described (34).

### Statistical Analysis

All analysed parameters were tested for normality of the data using the Kolmogorov-Smirnov test where possible. Differences between the groups were evaluated by Student’s t-test or ANOVA with Bonferroni *post-hoc* for normally distributed data or by Mann Whitney or Kruskal-Wallis for non-normally distributed data. Results are expressed as the mean ± the standard error of the mean (SEM) and values were considered statistically significant if p<0.05. Analyses were performed using Prism 5 software (GraphPad Software, La Jolla, CA, USA). Partial correlation analysis was performed to test the association between the presence of diabetes and placental protein expression adjusted for gestational age using SPSS 20.0 (IBM, USA).

## Results

### Signs of Placental Hypo-Perfusion and Increased Vascularisation Are Evident in T1D and GDM

**Figure 1:**
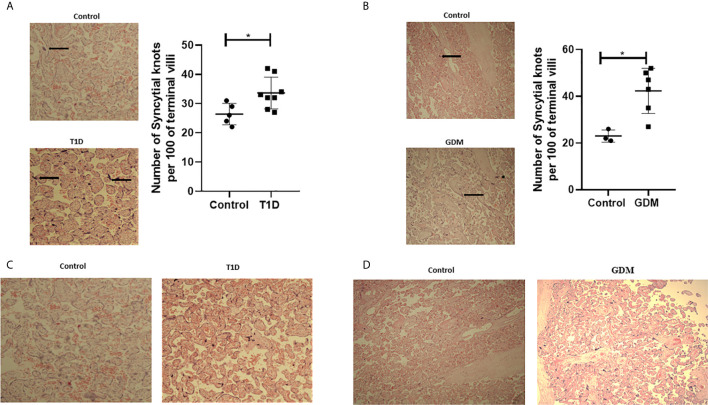
Signs of placenta hypoxia is evident concomitantly with increased levels of villous vascularity in diabetes. Paraffin-embedded placental sections were stained with H&E with 2 separate fields/section imaged at 4x magnification. The number of syncytial knots which are aggregates of syncytial nuclei at the surface of terminal villi (black spots indicated by arrows) are increased in placental samples from women with T1D **(A)** or GDM **(B)** compared to healthy controls (T1D, n=8, GDM, n=6 unpaired t-test, *<0.05). Villous vascularity (indicated by increased red staining) is abundant in T1D **(C)** and GDM **(D)** compared to healthy controls.

In order to assess placental morphology in pregnant women with diabetes, H&E staining was performed on placental sections and quantification of syncytial knots performed. Increased numbers of syncytial knots, aggregates of syncytial nuclei at the surface of terminal villi (42), can be indicative of the presence of placental hypoxia. However, advancing gestation is also consistent with an increased presence of syncytial knots. These structures can therefore be used to evaluate villous maturity (43). In normal placentae, approximately 30% of terminal villi have syncytial knots, whilst a higher percentage of syncytial knots is associated with uteroplacental malperfusion (44). In twelve out of fourteen placental samples from pregnant women with diabetes (T1D and GDM), an increased number of syncytial knots was observed compared to healthy pregnancies [**Figures 1** (**A**; T1D, n=8, p<0.05), **B**; GDM, n=6, p<0.05)]. Placental syncytial knots were positively correlated with T1D (r=0.619, p=0.024), which remained significant when adjusted for gestational age (r=0.66, p=0.02). Although syncytial knots were positively correlated with GDM (r=0.781, p=0.013), this correlation became non-significant when correcting to gestational age (r=0.459, p=0.252). Moreover, in all fourteen placental samples from diabetic pregnancies (T1D and GDM), a general increase in villous vascularity was observed, often associated with villous immaturity. This suggests that there is an increased number of capillaries and macrophages with the presence of fluid within the villous structure, which is a well-recognised feature of maternal diabetes (**Figures 1C**; T1D, **D**; GDM) (45). Furthermore, overstimulated placental angiogenesis can lead to impairment of the integrity of the vascular system and increased resistance to blood flow leading to hypoxia and the observed syncytial knots; consistent with the observed increased pro-angiogenic and reduced anti-angiogenic factors in these tissues (22). No differences in calcification were observed between diabetic and healthy placentae.

### Placental FKBPL and SIRT-1 Expression Is Reduced in T1D or GDM

Placental samples were collected from 14 pregnant women, six of whom developed GDM and eight of whom had pre-existing T1D. Control placental samples (n=8) were collected from healthy pregnant women matched for BMI and age, and foetal sex. Baseline characteristics are shown in **Table 1**. No statistically significant differences in age, parity, BMI, blood pressure, foetal sex or MOD were noted between any of the study groups compared to their matched controls. However, gestational age at delivery was lower in T1D and GDM patients compared to controls.

**Table 1:** Maternal baseline characteristics for pregnant women with type 1 diabetes and gestational diabetes.

	T1D (n=8)	Control (n=5)	p value
BMI	28.0 ± 6.9	25.9 ± 5.7	0.58
Age	24.6 ± 5.7	28.8 ± 4.8	0.20
sBP	115 ± 8.8	114 ± 8.7	0.92
dBP	73.8 ± 9.1	69.8 ± 24.4	0.36
Gestational age (weeks)	35.38 ± 0.8	39 ± 1.4	0.009
Foetal sex	2 females	1 female	0.85
	6 males	4 males	
Parity	5 Nulliparous	2 Nulliparous	0.8
	3 Primiparious or multiparous	3 Primiparous or multiparous	
Mode of delivery (MOD)	4 SVD	4 SVD	0.23
	2 assisted c/s	1 primary c/s	
	2 primary c/s		
	**GDM (n=6)**	**Control (n=3)**	**p value**
BMI	32.7 ± 8.9	29.34 ± 4.4	0.29
Age	31.8 ± 3.8	27 ± 2.6	0.06
sBP	122.1 ± 8.1	121.6 ± 1.8	0.9
dBP	85.3 ± 6.8	78.3 ± 8.1	0.16
Gestational age (weeks)	36.8 ± 1.2	39 ± 0	0.02
Foetal sex	2 females	3 males	0.17
	4 males		
Parity	2 Nulliparous	2 Nulliparous	0.29
	4 Primiparous or multiparous	1 Primiparous or multiparous	
Mode of delivery (MOD)	5 SVD	2 SVD	0.63
	1 primary c/s	1 primary c/s	

Data is presented as mean ± SD, two tailed paired Student’s t-test. Foetal sex was calculated by assuming female is zero and male is one. Mode of delivery was calculated by assuming SVD is zero, assisted c/s is one, and primary c/s is two. BMI, Body mass index; sBP- Systolic blood pressure; dBP, Diastolic blood pressure; SVD, spontaneous vaginal delivery; c/s, caesarean section.

**Figure 2:**
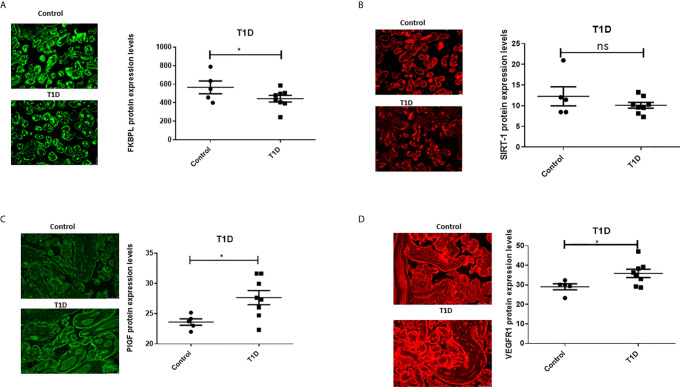
Placental angiogenic balance is disrupted in T1D. Placental slides from women with T1D *versus* age, BMI and foetal sex matched controls were stained with **(A)** FKBPL, **(B)** SIRT-1, **(C)** PlGF and **(D)** VEGF-R1 primary antibodies, followed by staining with green Alexaflour or red Cy3 secondary antibody. Six images per slide were taken at 20x magnification and the mean fluorescence quantified using Image J. Representative images inset. FKBPL/PlGF/SIRT-1: unpaired t-test, n=8; VEGF-R1: Mann-Whitney, *<0.05). ns, non-significant.

To assess the role of FKBPL and SIRT-1 in pregnancies complicated with diabetes, protein expression of FKBPL and SIRT-1 within placental samples collected from pregnant women with T1D or GDM was compared to controls, matched for age, BMI, and foetal sex. Our results demonstrated significant downregulation of FKBPL protein expression within placental sections of T1D patients compared to healthy controls (**Figure 2A**, n=8, p<0.05). FKBPL placental expression was negatively correlated with T1D (**Table 2**, r=-0.581, p=0.037), which remained significant when correcting for gestational age (**Table 2**, r=-0.65, p=0.022). SIRT-1 protein expression remained the same within the placental sections in the presence of T1D compared to controls (**Figure 2B**, n=8). No significant correlation was observed between placental SIRT-1 expression and T1D (**Table 2**, r=-0.428, p=0.145), however this correlation became borderline significant when adjusted for gestational age (**Table 2**, r=-0.578, p=0.049). Interestingly, PlGF protein expression was upregulated in placentae collected from women with pre-existing T1D (**Figure 2C**, n=8, p<0.05) with a similar pattern observed for VEGF-R1 protein (**Figure 2D**, n=8, p<0.05). PlGF expression in the placenta was positively correlated with T1D (**Table 2**, r=0.7, p=0.008), which remained significant when correcting for gestational age (**Table 2**, r=0.611, p=0.035). Similarly, VEGF-R1 expression was positively correlated with T1D with borderline significance (**Table 2**, r=0.551, p=0.051), however after adjusting for gestational age, this correlation was statistically significant (**Table 2**, r=0.643, p=0.024).

**Table 2:** Adjusted correlations for differences in gestational age between diabetic and healthy placentae.

Samples	FKBPL		SIRT-1		PIGF		VEGF-R1	
	Pearson correlation	Correlation controlled by GA	Pearson correlation	Correlation controlled by GA	Pearson correlation	Correlation controlled by GA	Pearson correlation	Correlation controlled by GA
T1D	r=-0.581	r=-0.65	r=-0.428	r=-0.578	r=0.7	r=0.611	r=0.551	r=0.643
	**p=0.037**	**p=0.022**	p=0.145	**p=0.049**	**p=0.008**	**p=0.035**	p=0.051	**p=0.024**
GDM	r=-0.059	r=-0.657	r=-0.964	r=-0.92	r=0.358	r=0.137	r=0.584	r=0.825
	p=0.88	p=0.077	**p=0.000**	**p=0.001**	p=0.345	p=0.746	p=0.098	**p=0.012**

Bold indicates statistical significance.

**Figure 3:**
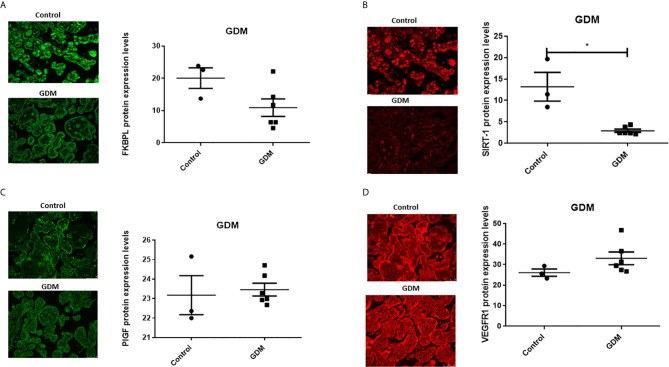
Placental angiogenic balance is disrupted in GDM. Placental slides from women with GDM versus age, BMI and foetal sex matched controls were stained with **(A)** FKBPL, **(B)** SIRT-1, **(C)** PlGF and **(D)** VEGF-R1 primary antibodies, followed by staining with green Alexaflour or red Cy3 secondary antibody. Six images per slide were taken at 20x magnification and the mean fluorescence quantified using Image J. Representative images inset. SIRT-1: Mann-Whitney test; n=6, *<0.05.

In the presence of GDM, placental FKBPL protein expression remained unchanged compared to healthy controls (**Figure 3A**, n=6, GDM; n=3, controls) with non-significant negative correlation (**Table 2**, r=-0.059, p=0.88), without further improvement after correcting for gestational age (**Table 2**, r=-0.657, p=0.077). GDM led to reduced SIRT-1 protein expression in the placenta (**Figure 3B**, n=6, p<0.05). SIRT-1 expression was negatively correlated with GDM (**Table 2**, r=-0.964, p=0.000), which remained significant after adjusting for gestational age (**Table 2**, r=-0.92, p=0.001). However, in contrast to T1D and similar to FKBPL, placental PlGF and VEGF-R1 protein expression remained unchanged in the presence of GDM compared to controls (**Figure 3**; C, PlGF; D, VEGF-R1). There was no significant correlation between PlGF expression and GDM (**Table 2**, r=0.358, p=0.345), which remained non-significant after correcting the values to gestational age (**Table 2**, r=0.137, p=0.746). Similarly, the correlation between VEGF-R1 expression and GDM within the placental sections was not significant (**Table 2**, r=0.584, p=0.098) however, there was a significant positive correlation once adjusted for gestational age (**Table 2**, r=0.825, p=0.012).

### FKBPL and SIRT-1 Protein Expression Are Downregulated in Response to High Glucose and Low Oxygen in ACH-3P Trophoblast Cells

**Figure 4:**
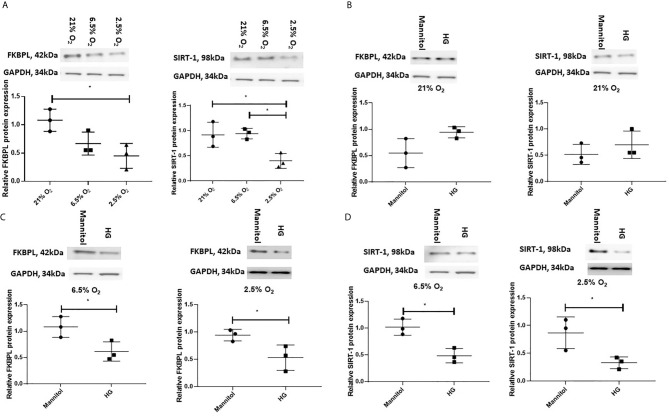
FKBPL and SIRT-1 protein expression is reduced in ACH-3P cells in high glucose conditions in the presence of low oxygen levels. ACH-3P cells were exposed to high glucose HG, (25mM) in the presence of varying oxygen (O_2_) conditions for 24 h before protein was extracted and Western Blotting performed. **(A)** FKBPL and SIRT-1 protein expression is reduced by low oxygen (2.5%) conditions at normal glucose concentration. **(B)** FKBPL and SIRT-1 protein expression remained unchanged in normal experimental oxygen conditions in the presence of high glucose compared to normal glucose. **(C)** FKBPL and **(D)** SIRT-1 protein expression was reduced in high glucose conditions at 6.5% and 2.5% O_2_ compared to normal glucose conditions at the same oxygen levels. Protein expression was normalised to GAPDH. (n=3, **(A)** one-way ANOVA with Bonferroni post-hoc test’ **(B–D)** unpaired t-test, *<0.05).

Having demonstrated that FKBPL and SIRT-1 are downregulated, in the presence of T1D or GDM within the placentae, respectively, collected following delivery, we wanted to investigate their regulation by diabetic environment early in pregnancy. For this purpose, we used a custom-made first-trimester trophoblast cell line, ACH-3P, generated by fusing primary first trimester trophoblasts with the choriocarcinoma cell line, AC1-1, so thereby closely resembling primary extravillous trophoblasts (40, 41). In order to mimic conditions associated with early pregnancy and placental development, ACH-3P cells were first exposed to varying oxygen (O_2_) concentrations (21%, 6.5% and 2.5%), similar to oxygen tension observed in the first trimester during placental development (6.5%, 2.5%) (46–48). To investigate the effect of a diabetic environment on FKBPL and SIRT-1 protein expression, ACH-3P cells were also exposed to high glucose (25 mM D-glucose) or normal glucose concentration (5 mM D-glucose + 20 mM Mannitol) in the presence of varying oxygen levels (21%, 6.5% and 2.5%) to mimic hyperglycaemic conditions in the first trimester of diabetic pregnancy. The effect of varying oxygen concentrations in ACH-3P cells was observed between 21% and 2.5% O_2_ in relation to downregulated FKBPL and SIRT-1 protein expression (**Figure 4A**, n=3, p<0.05), and between 6.5% and 2.5% O_2_ in relation to downregulated SIRT-1 protein expression (**Figure 4A**, n=3, p<0.05). FKBPL protein expression was not changed when ACH-3P cells were exposed to high glucose under normal experimental oxygen conditions (21% O_2_) (**Figure 4B**, n=3). Similarly, SIRT-1 protein expression also remained unchanged when ACH-3P cells were exposed to the same hyperglycaemic and normoxic conditions (**Figure 4B**, n=3). However, under low oxygen tensions of 6.5% (**Figure 4C**) and 2.5% O_2_ (**Figure 4D**) in high glucose environment', both FKBPL (n=3, p<0.05) and SIRT-1 (n=3, p<0.05) were downregulated, suggesting this effect is dependent on low oxygen conditions, consistent with hypoxia promoting a pro-angiogenic phenotype (49).

### FKBPL Plays a Key Role in Regulating Endothelial Cell Angiogenic Potential in Hyperglycaemia

**Figure 5:**
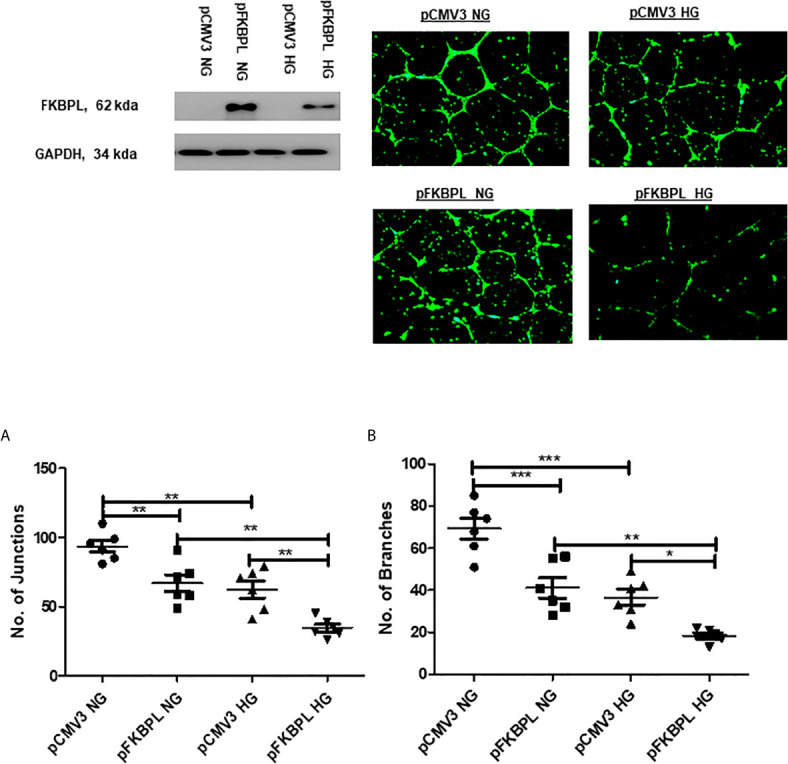
Angiogenic potential is further reduced with FKBPL overexpression in high glucose conditions in HUVECs. The number of **(A)** junctions and **(B)** branches was quantified following FKBPL overexpression in HUVECs for 24 h. The cells were stained with calcein stain, before being plated in Matrigel and exposed to high glucose (HG, 30 mM) or normal glucose (NG, 5.5 mM) media for 24 h. Six images/well were taken using a DMi8 microscope; representative images are shown inset. Images were analysed based on the number of junctions and branches formed using ImageJ angiogenesis macros. (n=6, *<0.05, **<0.01, ***<0.001, One-way ANOVA, followed by Bonferroni multiple comparison test).

To determine whether overexpression of FKBPL in hyperglycaemic and normal experimental oxygen (21% O_2_) conditions would restore normal angiogenesis in diabetic pregnancies, HUVECs were treated with a FKBPL overexpressing plasmid (pFKBPL) or empty vector control plasmid (pCMV3) for 24 h before being plated in Matrigel in the presence of high glucose media (30 mM) or normal glucose media (5.5 mM + 24.5 mM Mannitol) for 24 h. The number of junctions and branches formed was lower with FKBPL overexpression, consistent with its anti-angiogenic properties, in normal glucose conditions compared to empty vector control [**Figure 5**, n=6, p<0.01 (junctions), p<0.001 (branches)]. High glucose environment itself led to a similar reduction in endothelial cell angiogenic potential [(**Figure 5**, n=6, p<0.01 (junctions), p<0.001 (branches)]. Furthermore, when FKBPL was overexpressed in high glucose conditions, the number of junctions and branches was further reduced compared to empty vector high glucose control [(**Figure 5**, n=6, p<0.01 (junctions), p<0.05 (branches)]. In addition, the number of junctions and branches was lower with FKBPL overexpression in high glucose media compared to FKBPL overexpression in normal glucose media (**Figure 5**, n=6, p<0.01). This was performed at normal experimental oxygen conditions of 21%. High glucose and FKBPL overexpression appear to have additive effect on inhibition of angiogenesis.

## Discussion

Although differences at the molecular level between GDM and T1D placentae were demonstrated in our study, both diabetic phenotypes led to placental hypoxia or malperfusion. Previous study suggested that GDM and T2D show higher degree of uteroplacental malperfusion compared to T1D (50), which could be due to differences in sample sizes between the studies or different patient cohorts. In this study, we demonstrated that expression of the key proteins in placental angiogenesis, FKBPL, SIRT-1, PlGF and VEGF-R1 (31, 34, 51–53), is dysregulated in pregnancies complicated by diabetes with differential molecular changes observed between GDM and T1D. This is the first report to demonstrate a role for FKBPL in diabetic pregnancies, showing reduced expression of FKBPL in T1D only. We also show that PlGF and VEGF-R1 are upregulated in T1D, which represents an overstimulated but aberrant angiogenesis profile, also supported by placental histology results from diabetic pregnancies. Immune adaptation during pregnancy is essential to facilitate optimal conditions for foetal development and preparation for delivery and lactation (54). Given the fact that T1D is an autoimmune disease, several studies suggested that the immune adaptation is disturbed in pregnant women with T1D in addition to chronic inflammation, increasing the incidence of pregnancy complications including preeclampsia (55–57). As discussed in the introduction, FKBPL is a protein member of the immunophilin family with important roles in immune regulation (58). Furthermore, FKBPL was described recently as a regulator of inflammation and vascular integrity *via* modulating NF-kB signalling (59). Our results revealed that FKBPL expression is reduced in T1D placentae suggesting that FKBPL may also have a role in modulating immune adaptation and inflammation in this setting. This should be explored further in future studies. No changes in FKBPL, PlGF and VEGF-R1 protein expression were observed in GDM cohort, which could be due to a low number of samples, particularly controls, in this group (n=6, GDM and n=3, healthy controls) given that there was a trend observed with FKBPL and VEGF-R1.

While SIRT-1 expression was not affected by T1D, it was significantly downregulated in the presence of GDM. A large body of evidence suggests that SIRT-1 regulates glucose and lipid metabolism, in addition to inflammatory responses, gluconeogenesis, and the levels of reactive oxygen species, which together contribute to the development of insulin resistance (60–62). Previous studies showed that SIRT-1 expression or activity is reduced in people with T2D, GDM, or metabolic syndrome, which was also associated with endothelial dysfunction (63, 64). Our work also validates these previous findings that SIRT-1 is a key pathway affected by GDM (65). In addition, the role of SIRT-1 in trophoblast function, important for placental development has been demonstrated before (66). Taken together, SIRT-1 might have a role in improving placental development during GDM.

Furthermore, we demonstrated in relevant *in vitro* models that FKBPL and SIRT-1 proteins are reduced in both the presence of hyperglycaemia and lower oxygen tension (relevant for placental development) that could have adverse effects on trophoblast function and placental development (35). It is possible that oxidative stress could have a role in regulating FKBPL and SIRT-1 in these conditions and this should be explored in the future. This cell culture model in the presence of high glucose does not differentiate between these two phenotypes of diabetes (T1D and GDM), although hyperglycaemia in the first trimester of pregnancy is present more often in T1D. FKBPL showed a critical role in endothelial angiogenic potential in both normal and high glucose environment. This is important as the placenta is a highly vascularised organ with a crucial role in supporting growth and development of the foetus (19, 67). Controlled angiogenesis is key for normal placental development during pregnancy and any aberrant changes in angiogenic balance are closely associated with development of pregnancy complications such as preeclampsia. Dysregulation in FKBPL, SIRT-1, PlGF and VEGF-R1 demonstrated in this study, albeit with differential regulation in T1D or GDM, indicate the presence of angiogenic imbalance in pregnancies complicated by diabetes involving likely different mechanisms. Further studies should investigate how this affects trophoblast function and placental development.

Our previous study using FKBPL knockdown mice demonstrated strong pro-angiogenic phenotype with early signs of endothelial dysfunction (31). Findings from this study support an anti-angiogenic role of FKBPL in endothelial cells, however our data also suggest that low levels of endothelial FKBPL could be beneficial in diabetic pregnancies. This is because high levels of FKBPL appeared to cause further restriction of angiogenesis in hyperglycaemic conditions. This occurs despite reduced levels of FKBPL suggesting FKBPL overexpression involves other compensatory mechanisms. The effect of FKBPL overexpression on SIRT-1, VEGF-R1 and PlGF would have been useful to determine in these settings. Further investigations need to be conducted to better understand the mechanisms and effectsot FKBPL on the integrity of endothelial barrier in high glucose or diabetic environment. Moreover, SIRT-1, an important mediator of endothelial function, has been previously reported to be decreased in diabetes and associated with endothelial dysfunction (68), further supporting the presence of endothelial dysfunction in pregnancies complicated by diabetes potentially due to reduced levels of SIRT-1 or FKBPL. Hypoxia is a pro-angiogenic stimuli and the link between hypoxia and VEGF is well established (69). In pregnancies complicated by T1D or GDM, there is an increase in the expression levels of angiogenic growth factors perhaps as a result of compensatory angiogenesis. Vascular endothelial growth factor (VEGF) has been shown to increase as a result of hypoxia, which has the potential to impair integrity of the maternal and foetal vascular system (22, 70). Based on protein expression assays, higher levels of VEGF were detected in diabetic placentae compared to healthy placentae, indicative of hypervascularisation in diabetic placenta and suggesting that hypoxia is likely present in diabetic placentae (70, 71). This was confirmed in an *in vivo* model of GDM showing that hypoxia inducible factor-1α (HIF-1α) and VEGF levels were significantly higher in placentae in the presence of hyperglycaemia (72). As VEGF expression levels increase in the placenta in the presence of diabetes, angiogenesis and chorionic villous branching are stimulated offering larger surface area for nutrient uptake and allowing higher amounts of glucose to cross the placenta. Consequently, this leads to foetal hyperglycaemia and hyperinsulinemia (73). Furthermore, increased angiogenesis and chorionic villous branching can cause higher blood flow resistance and, potentially, higher maternal blood pressure that may lead to gestational hypertension and/or preeclampsia (22). Previous studies demonstrated that T1D and GDM can increase the risk of preeclampsia up to 4-fold (74–76). Our findings indicate that in diabetic placentae, there is an increase in syncytial knots indicative of hypoxia, in association with increased placental vascularity and immaturity. This could potentially be linked to increased PlGF and VEGF or decreased FKBPL or SIRT-1 expression as demonstrated in our study, promoting development of immature and leaky capillaries in the placenta (77). Considering that FKBPL was downregulated in T1D placentae, and that angiogenic potential was further reduced with FKBPL overexpression in high glucose conditions, this may suggest that low levels of FKBPL in diabetic placentae might be protective. However, the additive inhibitory effect on angiogenesis by high glucose and FKBPL overexpression appears to involve independent mechanisms. This needs to be explored further perhaps in the absence of growth factors within the media as these can also affect protein expression. We have previously shown in hypoxia (1% oxygen) that overexpression of FKBPL restores normal angiogenesis (34), which is not the case in high glucose conditions.

Trophoblast migration and invasion represent key processes driving placental development, particularly during remodelling of spiral uterine artery in pregnancy (78). We have previously shown that the FKBPL plasma levels are reduced before the onset of preeclampsia (34) and that low FKBPL levels lead to vascular dysfunction (31) hence downregulation of FKBPL in T1D may lead to vascular dysfunction and preeclampsia. This should be explored in the future. In this study, we demonstrated that FKBPL expression in ACH-3P first trimester trophoblast cells is downregulated by varying lower oxygen levels, recapitulating both normal and hypoxic conditions of the first trimester during placental development (35). The observed reduction in FKBPL levels could therefore play a role in promoting migration and invasion of trophoblast cells. We have previously shown that overexpression of FKBPL inhibited migration and invasion of cancer cells (25, 79). Considering that hyperglycaemia has the potential to decrease trophoblast invasion (80), FKBPL expression was found to be reduced in high glucose conditions, but only in the presence of low oxygen levels, which suggests that physiological normal levels of FKBPL may be important in determining primary trophoblast invasion during the first trimester of pregnancy in women with diabetes.

The limitations of this study include the small number of placentae and differences in gestational age between the groups. Nevertheless, using adjusted correlations we accounted for these limitations. We were unable to obtain information on medication for women with GDM, which is another limitation of our study. Nevertheless, we also demonstrated similar findings in terms of FKBPL and SIRT-1 regulation using our *in vitro* models of first trimester trophoblasts and endothelial cells in high glucose environment representative of diabetic pregnancies. However, our *in vitro* model cannot differentiate between GDM and T1D entirely. The effect of FKBPL overexpression in high glucose endothelial cell environment on other angiogenesis-related markers was not determined in this study.

In conclusion, placental FKBPL and SIRT-1 expression appears to be downregulated in response to diabetes in T1D and GDM, respectively, as well as following exposure to high glucose in trophoblast cells only in low oxygen conditions. This might suggest that carefully restoring FKBPL and SIRT-1 to normal physiological levels in diabetic conditions may reduce hyper-vascularisation early in pregnancy, with a potential to prevent subsequent pregnancy complications induced by impaired placental growth. The FKBPL’s effect and mechanism on endothelial cell angiogenesis, in association with placental growth, in high glucose environment needs to be deciphered further.

## Data Availability Statement

The raw data supporting the conclusions of this article can be made available from the corresponding author, upon reasonable request.

## Ethics Statement

The studies involving human participants were reviewed and approved by NHS Health Research Authority (ORECNI, 14/NW/1222) and the School of Medicine, Dentistry and Biomedical Sciences (Queen’s University Belfast). The patients/participants provided their written informed consent to participate in this study.

## Author Contributions

AA, NT, RM, K-AE, DJG, CJW, DJ, DO, SD, and LM designed the study, and/or recruited participants/collected samples, conducted experiments, analysed/interpreted the data, and wrote the manuscript. DH, AJH, VAH, DRM, ISY, TR, and GD contributed to the experimental design and/or conducted experiments and/or analysed/interpreted data. LM and DJG supervised AA. All authors contributed to the article and approved the submitted version.

## Funding

This work was supported by the PhD scholarship from the Hashemite University, Jordan and the Department for the Economy (DfE) - Global Challenge Research Fund (GCRF).

## Conflict of Interest

The authors declare that the research was conducted in the absence of any commercial or financial relationships that could be construed as a potential conflict of interest.
